# Efficient and Stable Perovskite Solar Cells based on Nitrogen‐Doped Carbon Nanodots

**DOI:** 10.1002/ente.202101059

**Published:** 2022-05-04

**Authors:** Silvia Collavini, Francesco Amato, Andrea Cabrera-Espinoza, Francesca Arcudi, Luka Đorđević, Ivet Kosta, Maurizio Prato, Juan Luis Delgado

**Affiliations:** ^1^ POLYMAT Avenida de Tolosa 72 and Faculty of Chemistry University of the Basque Country UPV/EHU P. Manuel Lardizabal 3 20018 Donostia-San Sebastián Spain; ^2^ Department of Chemical and Pharmaceutical Sciences INSTM UdR Trieste University of Trieste Via Licio Giorgieri 1 34127 Trieste Italy; ^3^ CIDETEC Basque Research and Technology Alliance (BRTA) P. Miramón 196 20014 Donostia-San Sebastián Spain; ^4^ CIC biomaGUNE Paseo de Miramón 182 20014 Donostia-San Sebastián Spain; ^5^ Ikerbasque Basque Foundation for Science 48013 Bilbao Spain

**Keywords:** carbon nanodots, perovskite solar cells, photovoltaics

## Abstract

The role of graphitic and amorphous nitrogen‐doped carbon dots (N‐CDs) as additives for perovskite solar cells (PSCs) is investigated. A detailed study of N‐CDs: perovskite (PVSK) blends through X‐ray diffraction, nuclear magnetic resonance, field emission scanning electron microscopy, UV–vis, and photoluminescence spectroscopy reveals the existence of interactions between N‐CDs and PVSK. The amorphous or graphitic nature of these carbon nanoforms, as well as the interactions between CDs and PVSK, clearly determines the photovoltaic outcome of the PSCs. Thus, a small amount of graphitic carbon dots (g‐N‐CDs) leads to more‐stable PSCs, while maintaining and even improving the power conversion efficiency (PCE). In addition, the long‐term evaluation of the g‐N‐CDs‐containing cells shows improvement of the PCE over time, up to 109% of the initial efficiency after 40 days while the reference performance is dropped to 86%.

## Graphical abstract

In this study, graphitic and amorphous nitrogen‐doped carbon dots are synthesized and characterized. Their role as additives for MA0.17FA0.83Pb (I0.83Br0.17)_3_ perovskite solar cells is investigated. The regular solar devices are characterized in terms of efficiency and stability, finding that the introduction of a small amount of graphitic carbon dots is beneficial for the stability of the cells.

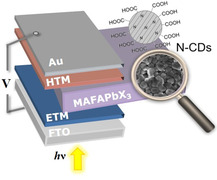

## 1 Introduction

Carbon dots (CDs) are nanosized (<10 nm) materials containing a carbon core and several surface functional groups, mainly hydroxyl and carboxylic groups.^[^ ^1^ ^]^ Similar to other carbon nanoforms,^[^ ^2^ ^]^ these materials have raised great interest thanks to a variety of appealing properties.^[^ ^3^ ^]^ In this regard, their ability to absorb in the UV–vis range and their high processability make them suitable candidates for photocatalytic applications.^[^ ^4^ ^]^

CDs have been recently employed as a replacement of the sensitizer in dye‐sensitized solar cells (DSSCs) or as additives in PSCs.^[^ ^1^ ^]^ The merging of the research fields of carbon dots and PVSK solar cells (PSCs) began in 2014, when Yang and co‐workers inserted a graphene quantum dots (GQDs) layer between the PVSK and the electron‐transporting material (ETM) TiO_2_.^[^ ^5^ ^]^ The recorded power conversion efficiency (PCE) of the GQDs‐containing PSCs was higher than that measured for the reference. Carbon quantum dots (CQDs) have been another type of carbon‐based material used in PSCs. The introduction of CQDs not only improves the morphology of the perovskite layer but also well adjusts the energy‐level matching of the perovskite film and the electrodes, promoting efficient carrier transfer.^[^ ^6^ ^]^ More recently, a similar experiment was carried out by Wang, Yang, and coworkers, who obtained a PCE of 19% using a blend of TiO_2_ and CDs.^[^ ^7^ ^]^ Song, Dai, and coworkers demonstrated that these CDs/PVSK blends could contribute to the improvement of the stability of the solar cells, which is a very important issue concerning PSCs.^[^ ^8^ ^]^ The CDs employed by these researchers showed the ability to convert UV light into blue light, providing PVSK with more light to harvest while protecting it from UV degradation.^[^ ^9^ ^]^ Fluorescent CDs properties can be tuned by functionalization to the extent that they can also be used as hole‐transporting materials (HTM) with success.^[^ ^10^ ^]^ In addition, the introduction of potassium‐functionalized CNDs@K directly into the PVSK layer demonstrated defect passivation ability, with a consequent improvement in the performance of the devices.^[^ ^11^ ^]^ As evidenced by a recent study by Zheng and coworkers, the doping and the surface composition of CDs may influence the energy levels of the films and the charge transfer processes in solar cells and light‐emitting diodes (LEDs).^[^ ^12^ ^]^

**Figure 1:**
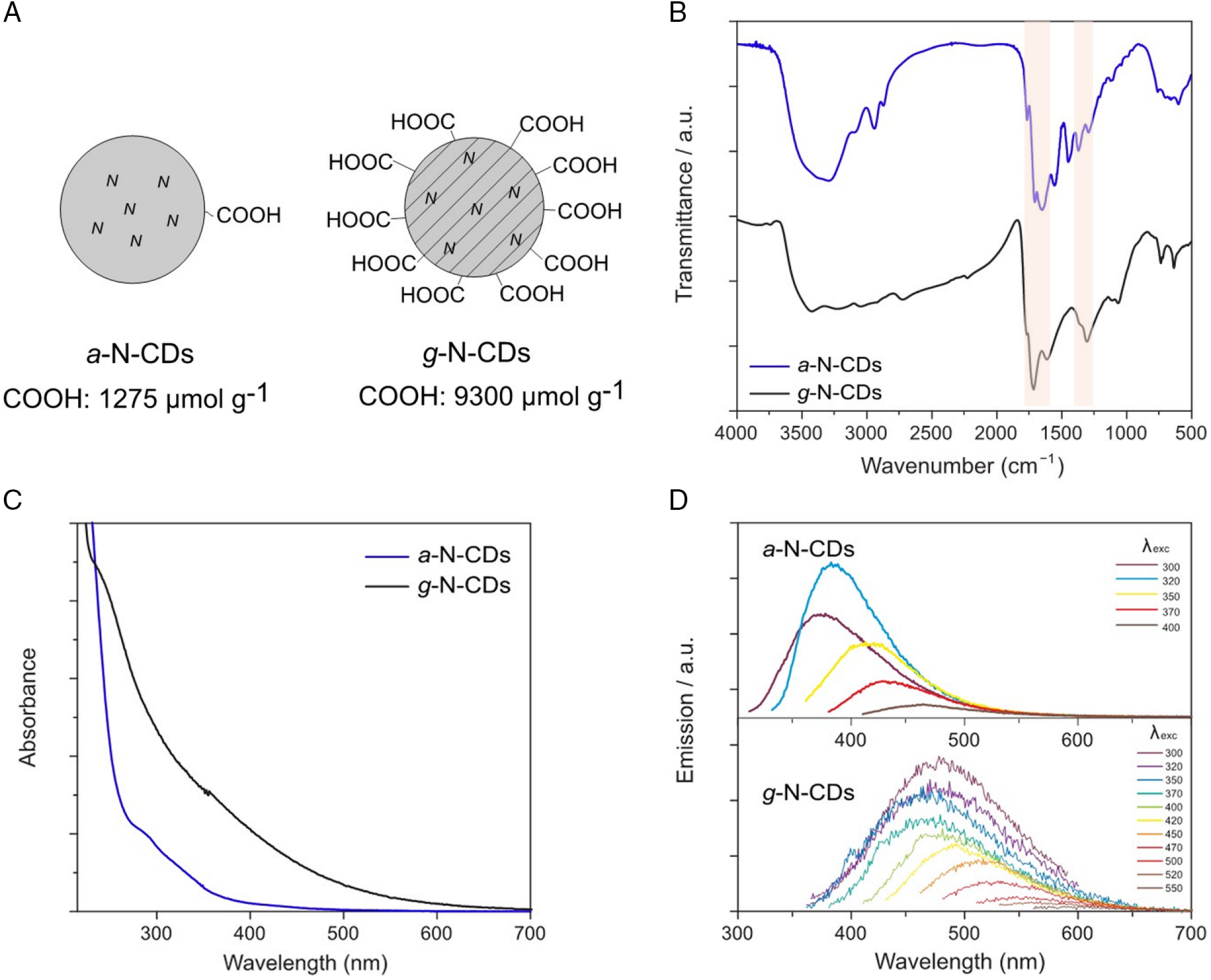
A) Schematic representation of the two carbon dots (CDs), highlighting the different amount of –COOH groups. B) Fourier transform infrared (FT‐IR) spectra of a‐N‐CDs (blue line) and g‐N‐CDs (black line) with the carbonyl stretching peaks highlighted. C) UV–vis spectra of a‐N‐CDs (blue line, 0.06 g L^−1^) and g‐N‐CDs (black line, 0.02 g L^−1^) in water at 298 K. D) Fluorescence emission spectra (at different excitation wavelengths) of a‐N‐CDs (0.06 g L^−1^) and g‐N‐CDs (0.02 g L^−1^) in water at 298 K.

In light of all these studies, we decided to prepare innovative CDs, namely amorphous and graphitic nitrogen‐doped CDs (a‐ and g‐N‐CDs, **Figure** **1**A) and investigate their effects on methylammonium (MA)‐formamidinium (FA) lead halide PVSK at different concentrations. As CDs are considered a core–shell type of nanoparticles, we focused on using CDs rich in carboxylic acids on their surface, to promote the beneficial ionic and hydrogen‐bonding stabilizing interactions with FA. In contrast, the effect that the amorphous or graphitic core plays on the stability of PSC was investigated. We proceeded to evaluate the effects on the device performance and, in addition, on the stability, which is the main issue regarding this kind of photovoltaic technology. Furthermore, we have tried to provide deeper insight into the mechanism of interaction between the N‐CDs and PVSK that lay behind the reported results.

## 2 Results and Discussion

### 2.1 Preparation and Characterization of the N‐CDs

The synthesis of amorphous or graphitic cores in N‐CDs requires different synthetic approaches. To obtain a‐N‐CDs, we used microwave‐assisted hydrothermal synthesis.^[^ ^13^ ^]^ This procedure is known to yield nanoparticles around 3 nm in size with an amorphous core and a surface rich in amine groups. These a‐N‐CDs were then subjected to a post‐synthetic modification with Meldrum's acid (2,2‐dimethyl‐1,3‐dioxane‐4,6‐dione) to change the surface functional groups to carboxylic acids (Scheme S1, Supporting Information). The successful modification was confirmed by the change in the Kaiser test results. After completion of the reaction, the amount of amino groups estimated was ≈75 μmol g^−1^, which was lower than that of the pre‐functionalized N‐CDs (≈1350 μmol g^−1^) and allowed us to estimate ≈1275 μmol g^−1^ of carboxylic groups on the surface of the a‐N‐CDs (Figure 1A). The chemical modification of the surface did not change considerably the photophysical properties (Figure S1–3, Supporting Information, for comparison between pre‐ and post‐modification). This is consistent with the change of the surface chemical composition slightly influencing absorption and emission spectra.^[^ ^14^ ^]^ In addition, the Fourier transform infrared (FT‐IR) spectroscopy spectrum of the post‐functionalized nanodots shows more pronounced features relative to the stretching modes of the carbonyl groups (1370, 1653, 1703, 1767 cm^−1^) as compared to the pristine nanodots (Figure S4, Supporting Information, for comparison between pre‐ and post‐modification) and C−N (1449 cm^−1^), C = N (1557 cm^−1^), C−H (2941 and 2871 cm^−1^), O−H/N−H (3292 cm^−1^) functional groups can be identified (Figure 1B).^[^ ^14^ ^]^

In the case of g‐N‐CDs, we used a synthetic procedure consisting in the thermal treatment in a muffle furnace of L‐aspartic acid in air at 320 °C for 100 h, which yields N‐doped carbon nanoparticles around 3 nm in size, exposing on the surface carboxylic acids. Heating to temperatures higher than 300 °C normally favors the formation of a graphitic carbon core, as previously reported.^[^ ^15^ ^]^ The FT‐IR spectrum shows that the primary surface functional groups are carbonyl groups (1308 and 1718 cm^−1^).^[^ ^15^ ^]^ The number of carboxylic functional groups in the g‐N‐CDs was estimated to be 9300 μmol g^−1^ (Figure 1A) by means of conductometric titration (performed by adding 15 mL of HCl 0.01 m to 17 mg of g‐N‐CDs, titrating with a solution of NaOH 0.01 m). Notably, the g‐N‐CDs are insoluble in H_2_O and the addition of NaOH is needed to solubilize this material in aqueous solutions or, alternatively, organic polar solvents like DMF are needed for analysis like fluorescence (FL) spectroscopy, as shown in Figure S5 and S6, Supporting Information.

The FT‐IR spectra of the two CDs show the typical features of carbonyl stretching (highlighted in Figure 1B). The graphitic CDs show higher molar absorption coefficients and improved absorption as compared to the amorphous ones due to the high *sp* ^2^ carbon content which is particularly significant at the tail into the visible region (Figure 1C for the spectra registered in water, Figure S7, Supporting Information, for those registered in DMF).^[14a]^ The FL spectra show an excitation wavelength dependence for both samples **(**Figure 1D), and the lower FL emission in the case of the graphitic one is consistent with an unpassivated surface.^[^ ^13^ ^]^

Figure S9, Supporting Information, depicts the X‐ray Diffraction (XRD) pattern of a‐N‐CD and g‐N‐CD. Both spectra showed peaks around 2*θ* = 28°, 43° and a weak peak at 53°, which are assigned to C(002) and C(100), C(004) of the hexagonal graphite structure (N‐CDs).^[^ ^16^ ^]^ In this regard, it is worth highlighting that the amorphous nature of a‐N‐CDs is represented by greater width peaks in comparison to the peaks of g‐N‐CDs.

### 2.2 Preparation and Characterization of N‐CDs: PVSK films

To obtain a good understanding of the possible N‐CDs‐PVSK interactions, we designed experiments containing a variety of N‐CDs concentrations. The g‐N‐CDs were blended with the PVSK precursor solution at different concentrations, in the range of 0.1–7.5 mg mL^−1^, while a‐N‐CDs in a concentration range of 0.1–20 mg mL^−1^, due to their higher solubility. Both were deposited by spin coating with the PVSK light‐harvesting layer.

The PVSK films were deposited from a precursor solution containing formamidinium iodide (FAI, 1.0 m), methylammonium bromide (CH_3_Br, 0.2 m), PbI_2_ (1.1 m), and PbBr_2_ (0.22 m) in anhydrous DMF:DMSO 4:1 (v:v), following a previously reported procedure.^[^ ^17^ ^]^ The chosen MA_0.17_FA_0.83_Pb(I_0.83_Br_0.17_)_3_ light‐harvesting layer will be referred to as PVSK from now on. The detailed fabrication procedure can be found in the Supporting Information.

**Figure 2:**
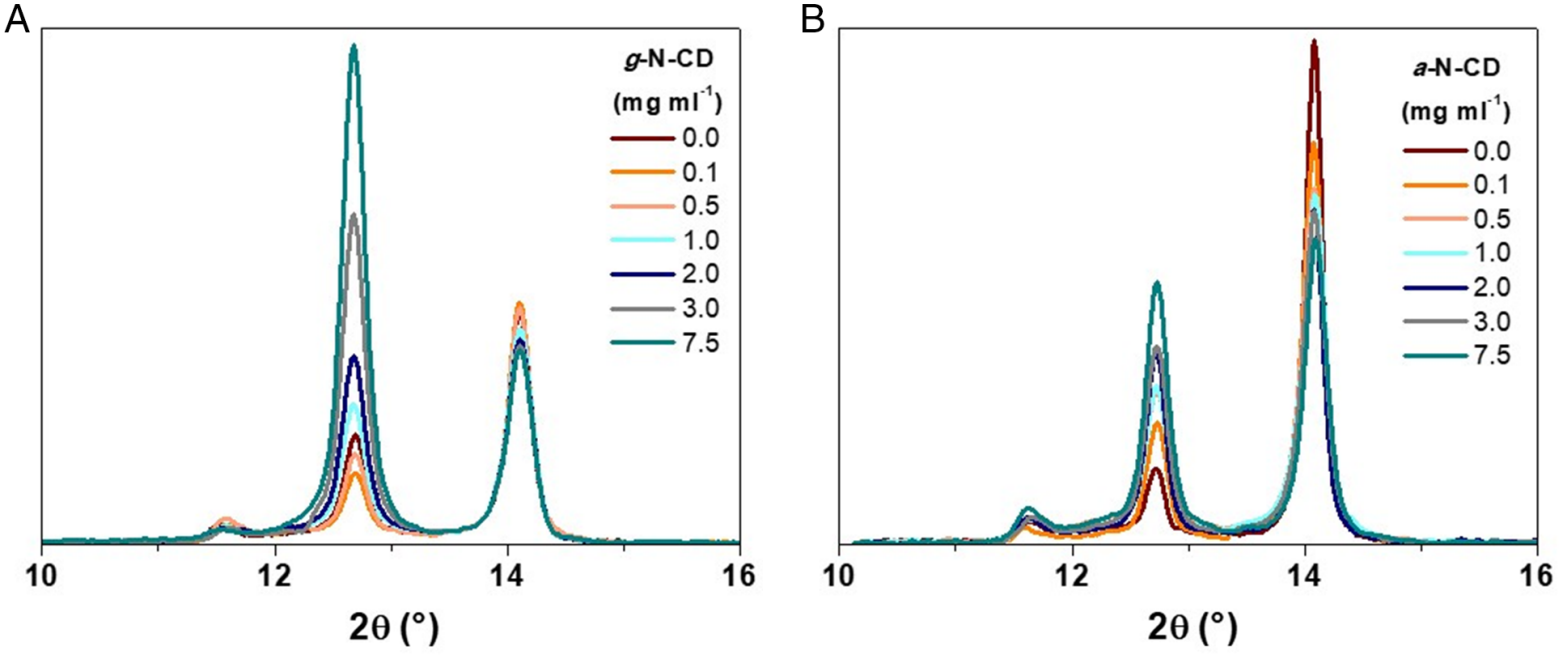
X‐ray Diffraction (XRD) analysis of the PVSK layers containing: a) g‐N‐CDs (Zoom 11‐16°), and B) a‐N‐CDs.

XRD measurements of g‐N‐CDs: PVSK showed in each and every case the typical PbI_2_ peak at ≈12.5°, whose intensity was compared to that of one characteristic PVSK peak at 14.0° (**Figure** **2** and S9, Supporting Information). The PVSK with 0, 0.1, and 0.5 mg mL^−1^ of g‐N‐CDs gave PVSK peaks with similar intensity, but both 0.1 and 0.5 mg mL^−1^ provided less intense PbI_2_ peaks compared to the reference. This means that the formation of the perovskite is favored by the presence of a small amount of g‐N‐CDs. In the case of a‐N‐CDs, all the investigated concentrations provided more intense PbI_2_ peaks, although this effect is less pronounced for 0.1 and 0.5 mg mL^−1^of a‐N‐CDs.

**Figure 3:**
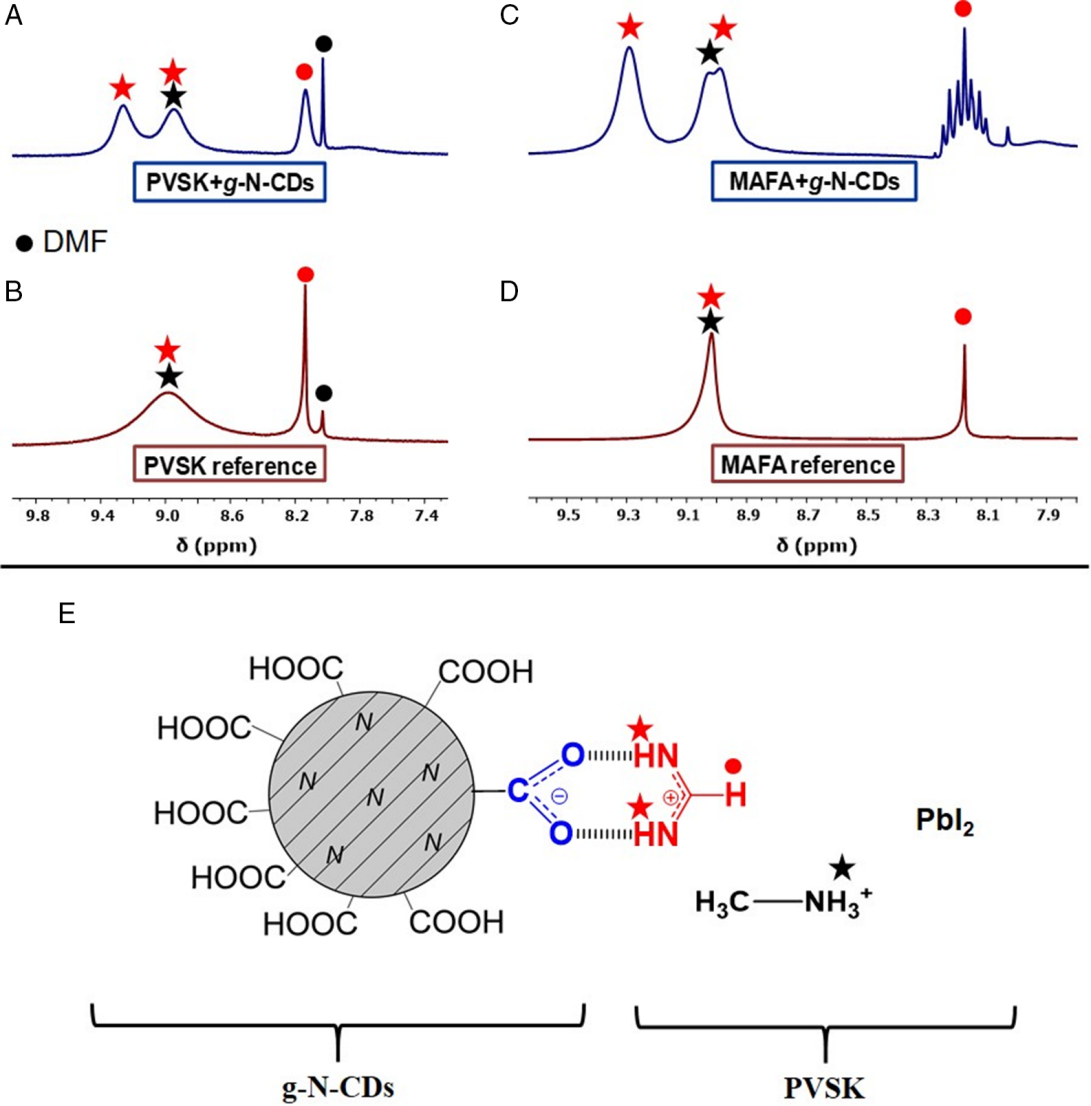
^1^H‐NMR spectra of: A) g‐N‐CD containing PVSK, B) PVSK reference, C) MA and FA cation mixture (MAFA) mixture with g‐N‐CD, D) MAFA reference mixture. All the spectra were registered on a DMF:DMSO 4:1 v/v solvent mixture. E) Scheme of all the possible interactions of g‐N‐CD with PVSK, including hydrogen bonds and ionic interactions.

To get a deeper understanding of the interactions between N‐CDs and PVSK, proton nuclear magnetic resonance (^1^H‐NMR) experiments were carried out. This technique is fundamental to understanding the chemical interactions between the PVSK and different additives.^[^ ^18^ ^]^ The spectrum of g‐N‐CDs‐containing PVSK was registered (DMF:DMSO 4:1 v/v) and compared to the pure PVSK reference dissolved in the same solvent. The spectra show some important differences: the signal of the protons bound to nitrogen in the amidinium moiety (around 9 ppm) splits and the proton bound to carbon in the same fragment (8.2 ppm) broadens. This effect might be due to an interaction between the FA cation and the carboxylic moieties of g‐N‐CDs, as previously observed with other carbon nanostructures.^[18a]^ The nitrogen protons of FA, interacting with the –COOH present in N‐CDs, are no longer equivalent, hence the splitting (**Figure** **3**A,B). This hypothesis is confirmed, through the comparison of ^1^H‐NMR spectra of MA and FA cation mixture (MAFA) registered with and without the addition of g‐N‐CDs. Similar to what was previously observed, there is a splitting of the FA signal in the presence of the N‐CDs. Furthermore, the CH signal shows a well‐resolved multiplicity given by the coupling with the now different sets of nitrogen protons (Figure 3C,D). The interactions suggested by these evidences are schematized in Figure 3E.

**Figure 4:**
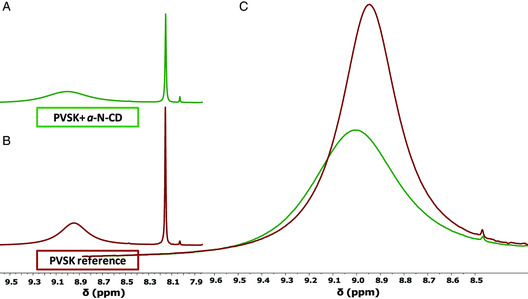
^1^H‐NMR spectra of: A) a‐N‐CD containing PVSK and B) PVSK reference. All the spectra were registered in a DMF: DMSO 4:1 v/v solvent mixture. C) Zoom of the spectra reported in A and B (region around 9 ppm).

The same NMR experiments were carried out considering a‐N‐CDs and PVSK reference (**Figure** **4**). In this case, the interaction between a‐N‐CDs and PVSK was not observed through NMR analysis, as evidenced in Figure 4. To explain this behavior, we need to consider the lower amount of the ‐COOH substituents, which should interact with the FA moieties (1275 μmol g^−1^ for a‐N‐CDs vs 9300 μmol g^−1^ for g‐N‐CD). Thus, the interaction between PVSK and a‐N‐CDs is more difficult to observe, and even if present, not detectable by NMR under these experimental conditions (Figure 4C).

**Figure 5:**
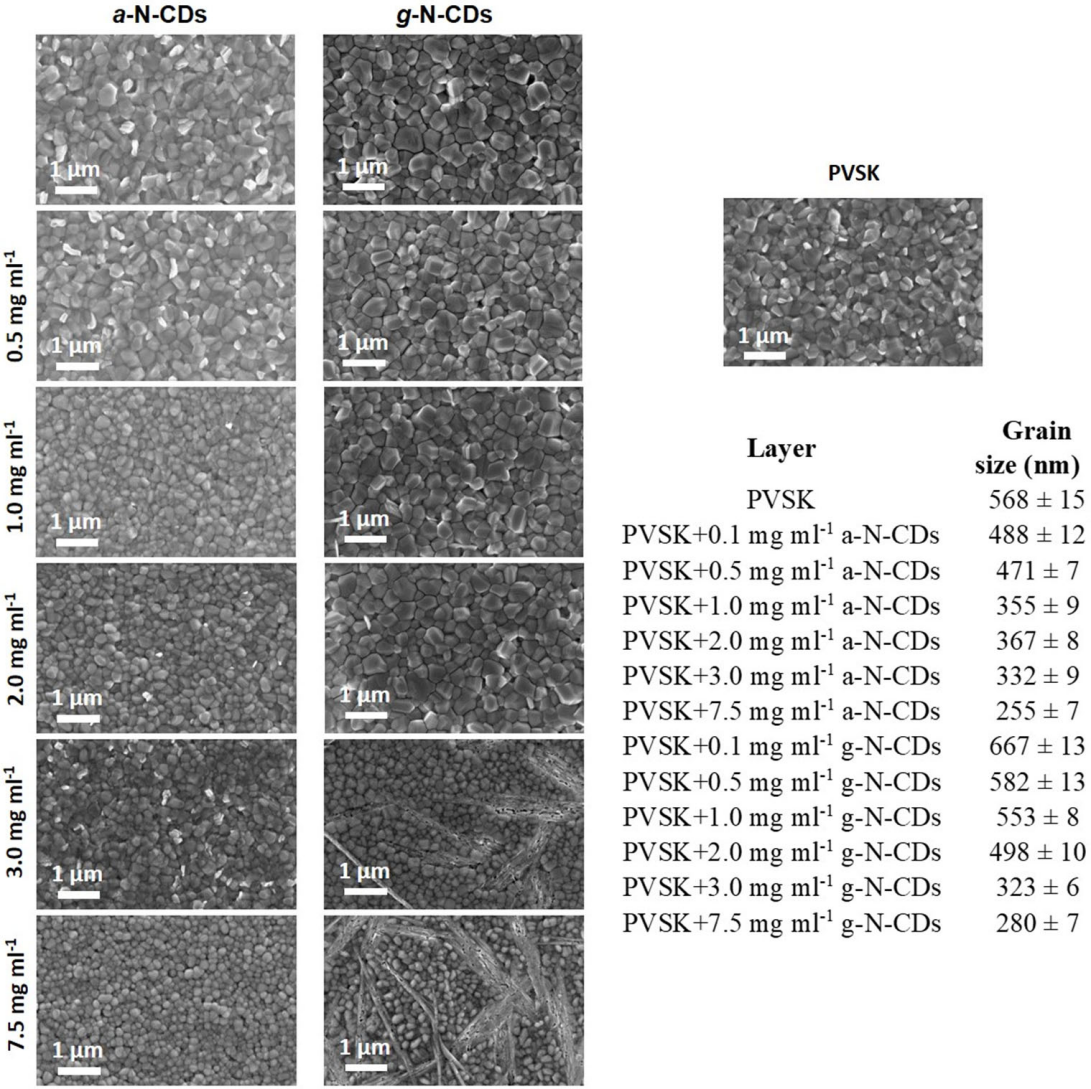
Top‐view field emission scanning electron microscopy (FE‐SEM) images of PVSK and PVSK containing different a‐ and g‐N‐CD concentrations. Average perovskite grain size of the a‐ and g‐N‐CD‐containing active layers.

Once we identify the existence of interactions between N‐CDs and PVSK, we proceed to study blends of these materials through electron microscopy. Thus, the field emission scanning electron microscopy (FE‐SEM) images of the PVSK films containing different concentrations of a‐ and g‐N‐CDs were registered (**Figure** **5**). The analysis of these scanning electron microscope (SEM) experiments shows how the average PVSK grain increases in size with 0.1 and 0.5 mg mL^−1^ of g‐N‐CDs, which is in agreement with that observed by XRD, indicating that g‐N‐CDs promote a better morphology in PVSK at these concentrations. The average PVSK grain size with 1 and 2 mg mL^−1^ of g‐N‐CDs is smaller than the reference PVSK; however, there is an important decrease in the grain size at 3 mg mL^−1^ of g‐N‐CDs, which is compatible with the observation of fiber‐like features observed in Figure 5. These fibers are most likely due to N‐CDs/N‐CDs interactions,^[^ ^19^ ^]^ and it can be observed that the amount of fibers increases with higher concentrations of g‐N‐CDs (7.5 mg mL^−1^). A similar study was performed with a‐N‐CD and it was found that the average PVSK grain size was smaller than the reference even at low concentrations (0.1 mg mL^−1^). This finding is in agreement with the XRD studies where every a‐N‐CD concentration increased the PbI_2_/PVSK ratio. The FE‐SEM micrographs of a‐N‐CD: PVSK films do not evidence the formation of visible fiber‐like aggregates even at high (3 or 7.5 mg mL^−1^) a‐N‐CD concentrations, consistent with the fact that amorphous nanoparticles lack the necessary patterns to interact between them. Nevertheless, the presence of higher concentrations of a‐N‐CD decrease the average grain size for the PVSK.

Cross‐sectional FESEM technique was used to obtain the thickness of the active layer of substrates based on fluorine‐doped tin oxide (FTO)/c‐TiO_2_ (113 ± 3 nm)/m‐TiO_2_/PSK + N‐CDs/Spiro‐OMeTAD (220 ± 5 nm). The results shown in Figure S10, Supporting Information do not display differences related to the amount of additive and the thickness of the perovskite layer, thus the thickness of the active layer remains constant in all the studied concentrations.

### 2.3 Preparation and Characterization of PSCs containing N‐CDs

**Figure 6:**
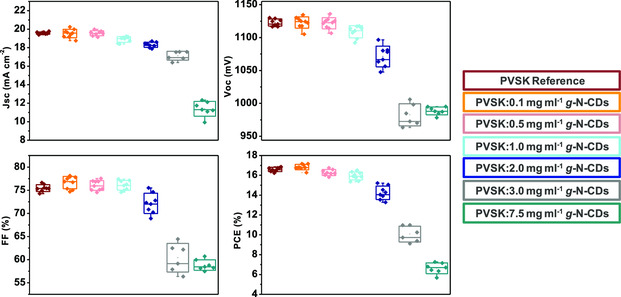
Box charts summarizing the different photovoltaic parameters, namely short‐circuit density (*J*_SC_), open‐circuit voltage (*V*_OC_), fill factor (*FF*), and power conversion efficiency (PCE), of the cells containing different concentrations of g‐N‐CDs.

**Figure 7:**
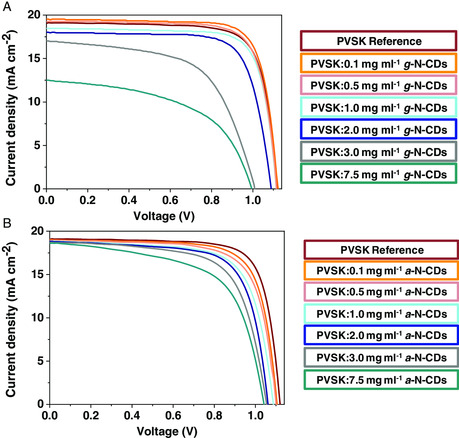
Best current density–voltage (*J–V*) curves for each: A) *g*‐N‐CD concentration and B) *a*‐N‐CD concentration.

Photovoltaic devices incorporating N‐CDs as additives in various amounts were fabricated following the regular architecture FTO/compact TiO_2_/mesoporous TiO_2_/MA_0.17_FA_0.83_Pb(I_0.83_Br_0.17_)_3_:N‐CDs /HTM/Au. The performances were evaluated by measuring their current density–voltage (*J–V*) curves. The parameters extracted from all the registered curves, short‐circuit density (*J*_SC_), open‐circuit voltage (*V*_OC_), fill factor (FF), and PCE, are summarized in the box charts reported in **Figure** **6**. A picture of the completed device is shown in Figure S11, Supporting Information. The best *J–V* curve for each concentration of g‐N‐CDs and a‐N‐CDs is reported in **Figure** **7**, and the photovoltaic parameters extracted from these curves are reported in Table S1 and S2, Supporting Information. As it can be appreciated in Figure 6, for g‐N‐CDs concentrations between 0.1 and 2 mg mL^−1^ the photovoltaic parameters are similar to those observed in the PVSK reference. This fact is in agreement with the results found by XRD, previously mentioned (Figure 2) and the average grain size found by FE‐SEM (Figure 5), as well as with the absorption and emission spectra evaluated for the perovskite layers with the different amounts of additives (Figure 9 and S15, Supporting Information).

Notably, at 0.1 mg mL^−1^, the average PCE (17.17%) is higher than the average PCE of the reference. This enhancement is due to a better morphology promoted by a higher value of average grain size, the absorption ability enhancement, and the improvement in charge extraction of the active layer, as will be discussed in more detail in the following.

As observed by FE‐SEM experiments (Figure 5), the addition of 0.1 mg mL^−1^ leads to an increase in the average grain size (667 nm for 0.1 mg mL of g‐N‐CDs vs 568 nm for reference perovskite). A concentration of 3.0 mg mL^−1^ of g‐N‐CDs produces fiber‐like aggregates and decreases the average grain size (323 nm for 3.0 mg mL^−1^ of g‐N‐CDs). This trend continues with the addition of higher amounts of g‐N‐CDs, resulting in an average gran size of 280 nm for 7.5 mg mL^−1^ of g‐N‐CDs and the worst PCE performance.

Regarding a‐N‐CDs, the photovoltaic parameters registered for the cells with the same concentrations used for g‐N‐CDs, and the corresponding statistics are reported in Figure S12, Supporting Information, together with the parameters summarized in Table S2, Supporting Information. The addition of these a‐N‐CDs produces in all the cases worse photovoltaic responses, which is in agreement with the average grain size decrease observed by FE‐SEM. Noteworthy the absence of aggregates, in this case, results in less intense changes for all the photovoltaic parameters. The registered currents were confirmed through external quantum efficiency (eQE) experiments, which are shown in Figure S14, Supporting Information, together with the comparison between the two series of values.

**Figure 8:**
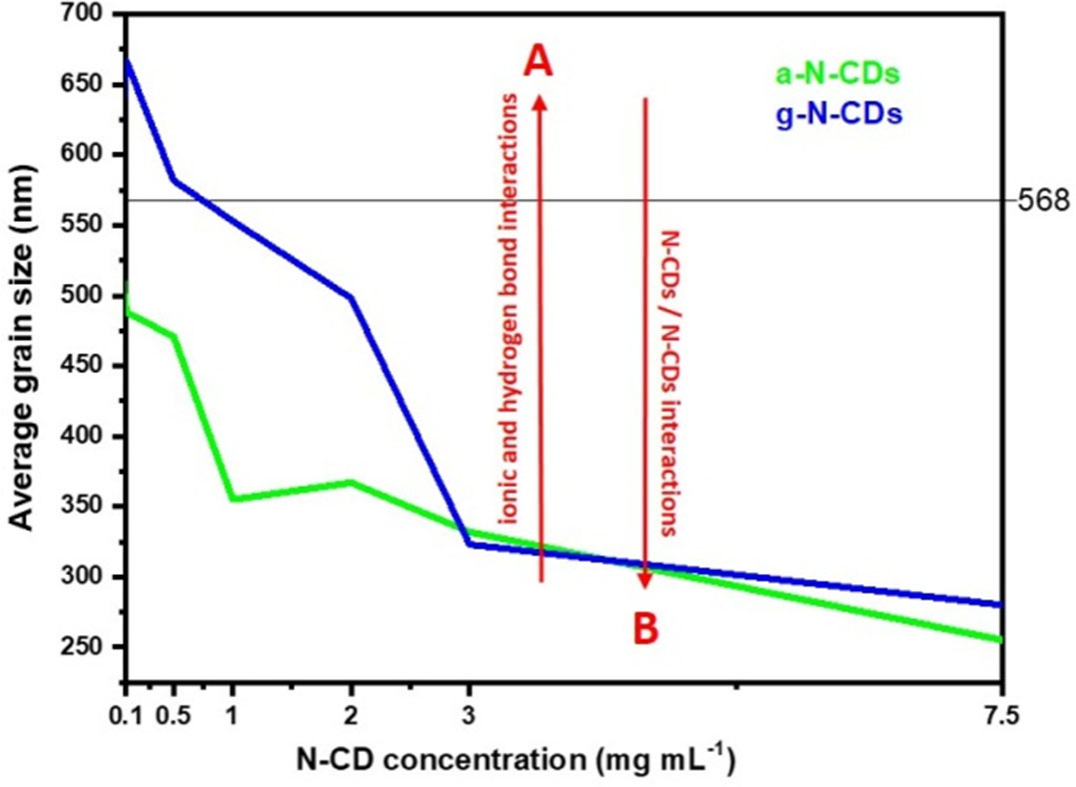
Average grain size of PVSK versus different amounts of N‐CDs.

As shown in **Figure** **8**, low concentrations of g‐N‐CD (0.1, 0.5, 1, and 2 mg mL^−1^) and a‐N‐CD (0.1 and 0.5 mg mL^−1^) produce PVSK grain size close to the reference value of 568 nm. However, at 3 mg mL^−1^ for g‐N‐CD and 1 mg mL^−1^ for a‐N‐CD there is a big decrease in PVSK grain size of around 150 nm (Figure 5). As evidenced by XRD, FE‐SEM, and ^1^H‐NMR, ionic and hydrogen bond interactions between N‐CDs and PVSK promote better crystallization, resulting in higher PVSK grain size and even higher PCE (0.1 mg mL^−1^ of g‐N‐CD). However when the concentration of N‐CDs increase, the N‐CDs/N‐CDs interactions takes control, inducing worse photovoltaic behavior due to a lower average of PVSK grain size. Interestingly, the point where the N‐CDs/N‐CDs interactions start ruling the morphology is related to the different amounts of COOH groups that these N‐CDs contain. Thus, in g‐N‐CDs, the concentration where N‐CDs/N‐CDs starts disturbing suitable PVSK grain size formation is around 3 mg mL^−1^, whereas this scenario occurs at lower concentrations of a‐N‐CD, owing to a 7.3 times lower amount of COOH groups.

Thus, we believe that at low N‐CDs concentrations, stabilizing ionic and hydrogen bond interactions between N‐CDs and PCSK (A) exist in a higher ratio than non‐stabilizing N‐CDs/N‐CDs (B) interactions, which are predominant at high N‐CDs concentrations.^[15b]^

**Figure 9:**
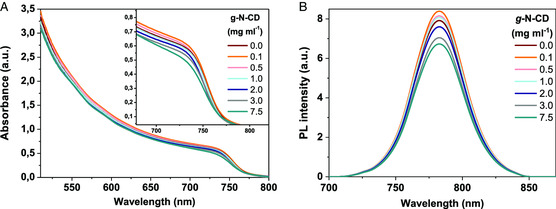
A) UV–vis absorption spectra of perovskite films with different concentrations of g‐N‐CD additive. B) Steady‐state photoluminescence (PL) spectra with different concentrations of g‐N‐CD additive.

The impact of the N‐CD additives on the optical properties of the perovskite layer was carefully studied by means of UV–vis and PL spectroscopy. As shown in **Figure** **9**A the light absorption intensities of perovskite films with g‐N‐CD concentration, increase slightly with 0.1 and 0.5 mg mL^−1^ as a consequence of the integrated effects of better crystallinity and larger sizes of perovskite grains. The enhanced UV–vis spectra of the perovskite layer with these amounts are in favor of a boosted *J*_SC._ Using a‐N‐CN (Figure S15A, Supporting Information), a decrease in the absorption intensity is observed when the concentration of additive increases, in agreement with the photovoltaic, XRD, and FE‐SEM results.

From the steady‐state PL spectra in Figure 9B, it is clear that the PL intensity of the perovskite film with 0.1 mg mL^−1^ is modesty higher than that of the pristine film, which is associated with an improvement in charge extraction and is related to the increase in *FF* (**Table** **1**). The PL spectra in Figure S15B, Supporting Information show a gradual decrease in PL intensity with increasing concentration of a‐N‐CDs, indicating that this type of additive apparently does not improve the extraction capacity of the perovskite layer.

**Table 1:** Best and average cell parameters of the g‐N‐CD‐containing devices

Devices	*J*_SC_ [mA cm^−2^]	*V*_OC_ [mV]	FF [%]	PCE [%]	Rs [Ω]	*R*_sh_ [× 103 Ω]
PVSK	19.8 19.58 ± 0.04	1130 1123 ± 2	76.6 75.5 ± 0.3	16.9 16.6 ± 0.1	9.4 ± 0.6	6.4 ± 0.2
PVSK + 0.1 mg ml^−1^ *g*‐N‐CDs	20.3 19.5 ± 0.2	1135 1123 ± 3	78.1 76.6 ± 0.4	17.17 16.84 ± 0.09	4.5 ± 0.9	9.6 ± 0.3
PVSK + 0.5 mg mL^−1^ *g*‐N‐CDs	20.0 19.6 ± 0.1	1136 1122 ± 3	77.5 76.0 ± 0.4	16.8 16.3 ± 0.1	7.8 ± 0.7	6.9 ± 0.3
PVSK + 1.0 mg mL^−1^ *g*‐N‐CDs	19.2 18.9 ± 0.1	1118 1109 ± 3	77.4 76.1 ± 0.4	16.5 15.9 ± 0.1	17.3 ± 0.8	6.3 ± 0.2
PVSK + 2.0 mg mL^−1^ *g*‐N‐CDs	18.7 18.3 ± 0.1	1097 1071 ± 6	75.5 72.1 ± 0.8	15.2 14.2 ± 0.2	24 ± 1	4.7 ± 0.4
PVSK + 3.0 mg mL^−1^ *g*‐N‐CDs	17.6 17.1 ± 0.2	1006 983 ± 7	64 60 ± 1	11.0 10.1 ± 0.3	40 ± 4	2.0 ± 0.2
PVSK + 7.5 mg mL^−1^ *g*‐N‐CDs	12.3 11.4 ± 0.3	995 989 ± 2	60.7 58.8 ± 0.4	7.3 6.6 ± 0.2	65 ± 3	0.9 ± 0.2

### 2.4 Stability Experiments

**Figure 10:**
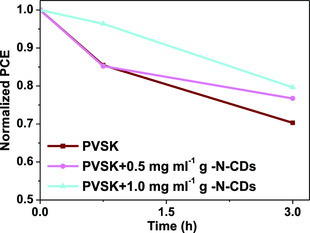
Short‐term thermal stress test of 0.5 and 1.0 mg mL^−1^ g‐N‐CD‐containing devices compared to the PVSK reference. Three devices were tested for each condition.

Considering that the best PCE data were obtained for g‐N‐CDs containing devices, we focused on the study of the stability of these devices. Therefore, the short‐term stability of g‐N‐CDs‐containing solar cells was evaluated. This experiment was carried out by leaving devices containing different amounts of g‐N‐CDs additives under one sun illumination at 80 °C, and at ambient air conditions (H_R_: 50%, oxygen). The cells were left under these stress conditions for 3 h and their performances were measured at the beginning, after 1 h, and at the end of the experiment. As it can be observed in **Figure** **10**, there is a positive effect on the stability due to the presence of the g‐N‐CDs additive. After 3 h, the PVSK reference retained 70% of its initial efficiency, while solar cells containing 0.5 and 1.0 mg mL^−1^ of g‐N‐CDs retained respectively, 77% and 79% of the initial efficiency (Figure 10).

**Figure 11:**
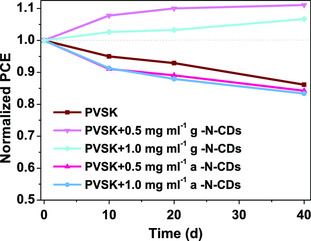
Long‐term stability test of 0.5 and 1.0 mg mL^−1^ a‐ and g‐N‐CD‐containing devices with respect to their PVSK reference. Three devices were tested for each condition.

Inspired by the results obtained on this preliminary test, long‐term stability of the solar cells containing g‐N‐CDs was performed (**Figure** **11**). This experiment was carried out by leaving the devices in a drawer, and exposed to the ambient atmosphere (i.e., in presence of oxygen and water). After the first 10 days, both g‐N‐CDs‐containing devices showed improvement in the performances, while the performances of the reference and a‐N‐CD‐containing cells dropped. After 20 days the performances of the reference and a‐N‐CD‐containing cells were decreasing slowly, while those of the g‐N‐CDs‐containing cells kept increasing. At the end of the experiment, cells with 0.5 and 1.0 mg mL^−1^ of g‐N‐CDs had their performances increased to 109% and 106%, respectively, while the reference dropped to 86% of its initial efficiency.

The observed PCE enhancement is mainly due to a progressive increase of *V*_OC_ of the solar cells containing g‐N‐CD, during the development of the experiment (Figure S16, Supporting Information). This observation points toward a spontaneous increase of PCE probably caused by a reduction of strain in perovskite films,^[^ ^20^ ^]^ due to the ionic and hydrogen bond interactions between g‐N‐CDs and PVSK.

Cells with lower g‐N‐CD concentrations were also tested, but their behavior was the same as the reference's (Figure S17, Supporting Information).

Short‐term stability of devices including a‐N‐CDs was also tested under the same conditions used in the previous experiment. As shown in Figure S18, Supporting Information, the a‐N‐CD‐containing PVSK devices suffered the stress test more than the PVSK reference. The less strong interactions between a‐N‐CDs and PVSK might be the reason why the devices containing these additives are less stabilized than the g‐N‐CD‐containing devices.

## 3 Conclusions

Graphitic and amorphous N‐CDs have been synthesized and fully characterized by means of XRD, UV–vis, FL emission, and FT‐IR spectroscopies. A complete study of N‐CDs : PVSK films by means of XRD, NMR, FE‐SEM, UV–vis spectroscopy, and PL spectroscopy, evidences the existence of interactions between N‐CDs and PVSK. These carbon nanoforms have been incorporated into PSCs at different concentrations, and the optimum amount of N‐CDs to achieve efficient devices has been determined. Importantly, the amorphous or graphitic nature of these nanoforms clearly determines the photovoltaic outcome of the PSCs, while ionic and hydrogen bond interactions between N‐CDs and PVSK are the driving force of the observed stabilization. In fact, in every case, the stability of the g‐N‐CDs‐containing cells was improved. Moreover, the long‐term evaluation of the performances of the cells showed an improvement in the PCE of the g‐N‐CDs‐containing cells over time, up to 109% of the initial efficiency after 40 days while the reference performance dropped to 86%.

## Conflict of Interest

The authors declare no conflict of interest.

## Supporting information

Supplementary Material

## Data Availability

The data that support the findings of this study are available from the corresponding author upon reasonable request.
