# Supplementary material for: Efficient and Stable Perovskite Solar Cells based on Nitrogen‐Doped Carbon Nanodots
Source: Energy Technol (Weinh). 2022 May 4;10(6):2101059. doi: 10.1002/ente.202101059 (PMC9286678; doi:10.1002/ente.202101059)
Supplement: Supplementary file 1 — Supplementary Material [file ENTE-10-0-s001.pdf]

Efficient and Stable Perovskite Solar Cells based on Nitrogen-Doped Carbon Nanodots

Silvia Collavini [1], Francesco Amato [2], Andrea Cabrera-Espinoza [1], Francesca Arcudi [2], Luka Đorđević [2], Ivet Kosta [3], Maurizio Prato [2,4,5], and Juan Luis Delgado.[1,5]**

1 POLYMAT, University of the Basque Country UPV/EHU, Avenida de Tolosa 72 & Faculty of Chemistry, P. Manuel Lardizabal 3, 20018 Donostia-San Sebastián, Spain.
2 Department of Chemical and Pharmaceutical Sciences, INSTM UdR Trieste, Via Licio Giorgieri 1, University of Trieste, 34127 Trieste, Italy.
3 CIDETEC, Basque Research and Technology Alliance (BRTA), P. Miramón 196, 20014 Donostia-San Sebastián, Spain.
4 CIC biomaGUNE, Paseo de Miramón 182, 20014 Donostia-San Sebastián, Spain.
5 Ikerbasque, Basque Foundation for Science, 48013 Bilbao, Spain.
E-mail: prato@units.it ; juanluis.delgado@polymat.eu

Methods and Materials

CDs Preparation and Characterization

Pristine *a*-N-CDs were synthesized according to a previously reported method,[1] and *g*-N-CDs were synthesized according to a reported procedure.[2]

Meldrum's acid (2,2-dimethyl-1,3-dioxane-4,6-dione), 4-dimethylaminopyridine, Kaiser test kit and Sephadex LH−20 were purchased from Sigma-Aldrich. Dialysis membrane (Float-A-Lyzer, MWCO 0.5-1 KDa) were bought from Spectrum Labs. Anhydrous *N*,*N*-dimethylformamide (DMF) was purchased at Acros organics and used without any purification.

Synthesis of a-N-CDs

The synthesis of *a*-N-CDs with carboxylic acid groups on the surface was accomplished by post-functionalization of pristine *a*-N-CDs with amino group on the surface (*a*-N-CDs-NH_2) with Meldrum's acid (2,2-dimethyl-1,3-dioxane-4,6-dione). Pristine *a*-N-CDs (25.0 mg, 0.034 mmol amines) were dissolved in 5 mL of anhydrous DMF, then Meldrum's acid (24.5 mg, 0.17 mmol) and 4-dimethylaminopyridine (2.5 mg, 0.020 mmol) were added. The mixture was stirred under argon at 80°C for 48 h. The volatiles were removed under high vacuum, the residue was dissolved in methanol and the purification was accomplished through size exclusion chromatography using Sephadex LH-20.[3] The residue was taken up in water and lyophilized.

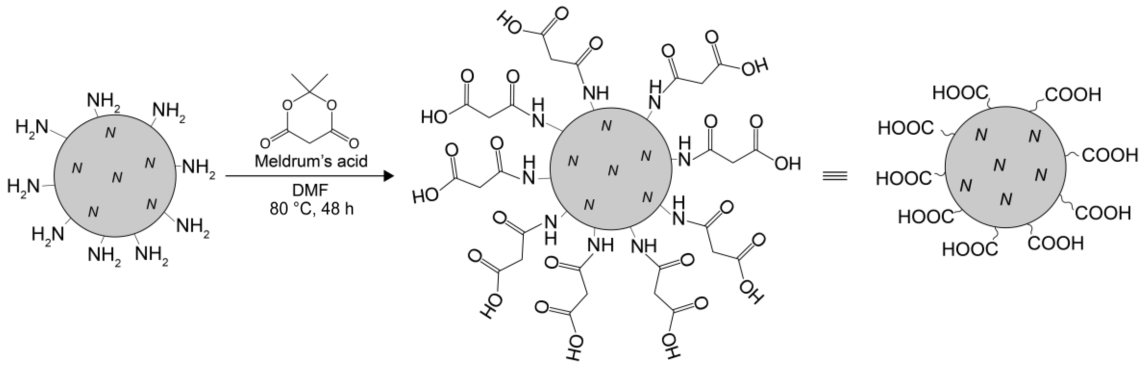

Scheme S1: Synthesis of the target *a*-N-CDs with carboxylic acid groups on the surface starting from the pristine *a*-N-CDs with amino groups on the surface.

Fourier-transform Infrared (FT-IR) spectra (KBr) were recorded on a Perkin Elmer 2000 spectrometer. UV–Vis spectra were recorded on a PerkinElmer Lambda 35 UV–Vis spectrophotometer and baseline corrected. Fluorescence spectra were recorded on a Varian Cary Eclipse fluorescence spectrophotometer.

PSCs Preparation and Characterization

The materials used in the photovoltaic study were obtained from commercial suppliers in high purity and used without further purification: glass/FTO (Nippon Sheet Glass), methylammonium iodide (MAI, DYESOL), FAI (DYESOL), $PbBr_2$ (99.99%, TCI chemicals), PbI_2 (99.99%, TCI chemicals), $TiCl_4$ (Sigma-Aldrich), TiO_2 paste (DYESOL), spiro-OMeTAD (Solarpur), lithium bis(trifluoromethane) sulfonimidate (LiTFSI, 99.9%, Sigma-Aldrich), tert–butylpyridine (96%, Sigma–Aldrich), tris(2-(1*H*-pyrazol-1-yl)-4-*tert*-butylpyridine)cobalt(III) tri[bis(trifluoromethane)sulfonimide] (FK 209, Dyenamo), DMF (extra dry, Acros Organics), DMSO (extra dry, Acros Organics), acetone (technical grade, Scharlab), EtOH (technical grade, Scharlab), chlorobenzene (extra dry, Acros Organics) and acetonitrile (extra dry, Acros Organics).

The ^{1}H-NMR spectra (DMF:DMSO 4:1) were taken on a Bruker NMR spectrometer (300 MHz) at room temperature. Chemical shifts (δ) are reported in ppm. The concentration of the PVSK NMR solutions was the same as that of the perovskite solution, with a CD concentration of 1 mg ml^{-1} for the g-N-CDs and 10 mg ml^{-1} for the a-N-CDs. For the MAFA NMRs the concentration was 1M with the same relative amount of g-N-CDs.

The morphologies and structural properties of the films were analyzed with an ULTRA plus ZEISS field-emission scanning electron microscope (FESEM) and a Bruker AXS-D8 Advance X-ray diffractometer with CuKa radiation.

The perovskite films for UV-Vis and photoluminescence spectroscopy were prepared by spin-coating of the precursor solutions on well-cleaned quartz glass substrates inside a glovebox and using the same conditions for the preparation of active layer in the solar cells. The exciting wavelength used was 507 nm, with a slit width of excitation and emission of 15 nm. The measurements were made in the same sensitivity of photomultiplier and same scan speed (200 nm/min).

The J–V characteristics were measured using a 450 W xenon light source (Oriel). The light intensity was calibrated with a Si photodiode equipped with an IR-cutoff filter (KG3,

Schott) and it was recorded during each measurement. Current-voltage characteristics of the cells were obtained by applying an external voltage bias while measuring the current response with a digital source meter (Keithley 2400). The voltage scan rate was 20 mV s^{-1}, and no device preconditioning was applied before starting the measurement, such as light soaking or forward voltage bias applied for a long time. The cells were masked with a black metal mask of 0.16 cm^2 to estimate the active area and reduce the influence of the scattered light.

Substrate and electron-transporting layer preparation:

FTO-glass substrates (10 Ω sq-1) were cleaned by ultrasonication in deionized water with soap (2% Hellmanex water solution) for 15 min. After thorough rinsing with deionized water, the substrates were further ultrasonicated with EtOH and acetone for 30 minutes each. Finally, the substrates were dried under a nitrogen flow and, in order to eliminate any organic impurity, they were further treated in an UV-ozone cleaner for 15 min. Afterwards, a TiO_2 compact layer was deposited on FTO via spin-coating for 20s at 5000 rpm from a precursor solution of titanium chloride 2M in water. After the spin-coating procedure, the substrates were left at 100 °C for 10 min. Then, the mesoporous TiO_2 layer was deposited by spin coating for 10 s at 4000 rpm with a ramp of 2000 rpm s-1, using 30 nm particle paste diluted in EtOH to achieve 150-200 nm thick layer. Subsequently, the substrates were sintered following a heating ramp up to 450 °C, at which they were left for 30 min under dry air flow. The mesoporous TiO_2 was doped by spin-coating of a solution of Li-TFSI in CH_3CN (10 mg mL^{-1}) at 3000 rpm for 10 s. Finally, the electrode with Li-doped mesoporous TiO_2 was completed with a second sintering process, the same as before. After cooling down to 150 °C, the substrates were immediately transferred to a nitrogen-filled glove box for the deposition of the perovskite films.

Perovskite precursor solution and film preparation

The perovskite films were deposited from a precursor solution containing FAI (1 M), PbI_2 (1.1 M), MABr (0.2 M) and $PbBr_2$ (0.22 M) in anhydrous DMF:DMSO 4:1 (v:v) solvent, following a previously reported procedure.[4] The chosen amount of *g*-N-CDs (0.1, 0,5, 1, 2, 3, or 7.5 mg ml^{-1}) was added to the solvent before the dissolution of the perovskite components. The perovskite solution was spin coated in a nitrogen-filled glove box through a two-step program at 1000 and 5000 rpm for 10 and 20 s, respectively. During the second step, 150 μL of chlorobenzene were poured on the spinning substrate 5 s before the end of the program. The substrates were then annealed at 100 °C for 45 min in a nitrogen-filled glove box.

Hole transporting layer and gold contact deposition

After the perovskite annealing the substrates were cooled down for few minutes and 50 μl spiro-OMeTAD solution (70 mmol in chlorobenzene) were spun at 4000 rpm for 20 s. Before deposition, this solution was doped with *tert*-butyl pyridine (3.3 mol/mol with respect to Spiro), Bis(trifluoromethane)sulfonimide lithium salt (1.8 M in acetonitrile, 0.5 mol/mol with respect to Spiro) and FK209 (0.25 M in acetonitrile, 0.05 mol/mol with respect to

Spiro). Finally, an 80 nm-thick gold electrode was thermally evaporated through an appropriate shadow mask on top of the hole transporting layer to produce a completed PSC device.

Supporting Figures

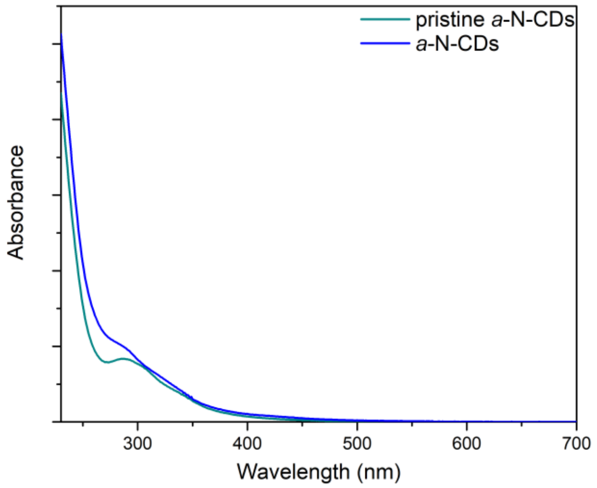

Figure S1. UV-Vis spectra of pristine *a*-N-CDs with an amino-rich surface (green line) and *a*-N-CDs with carboxylic acid groups on the surface (blue line) in water at 298 K.

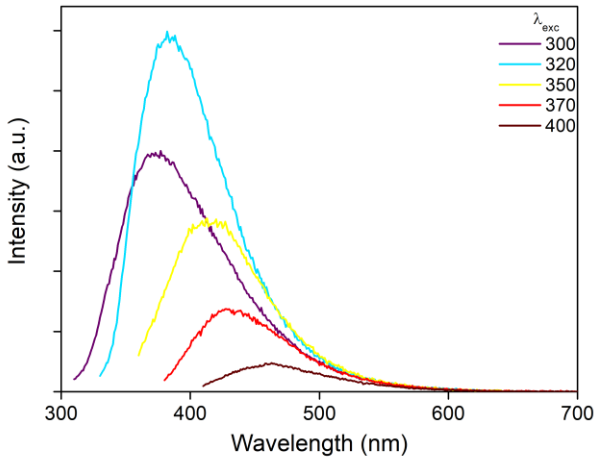

Figure S2. FL emission spectra (at different excitation wavelengths) of *a*-N-CDs with carboxylic acid groups on the surface in water at 298 K.

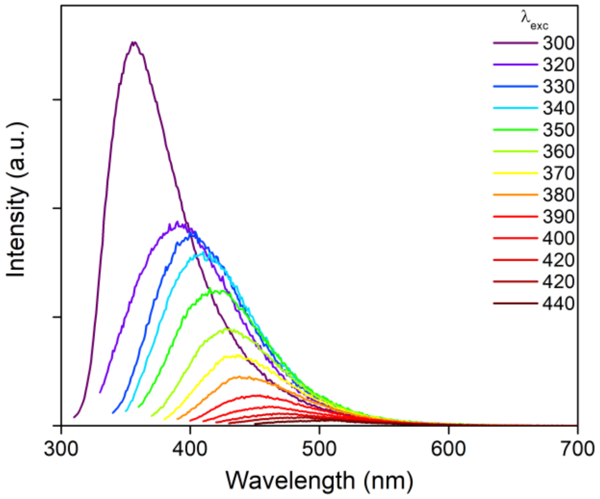

Figure S3. FL emission spectra (at different excitation wavelengths) of pristine *a*-N-CDs with an amino-rich surface in water at 298 K.

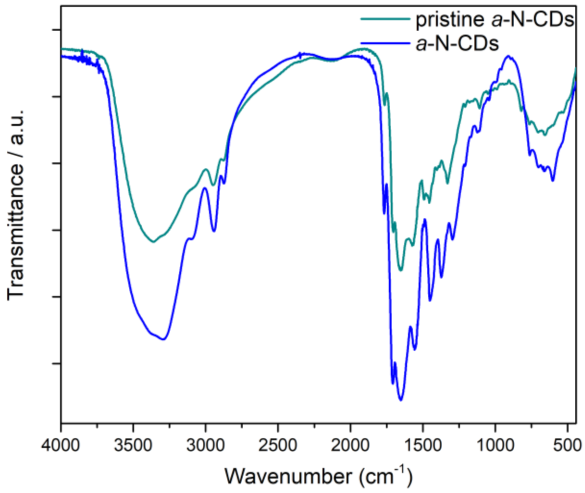

Figure S4. FT-IR spectra of pristine *a*-N-CDs with an amino-rich surface (green line) and *a*-N-CDs with carboxylic acid groups on the surface (blue line).

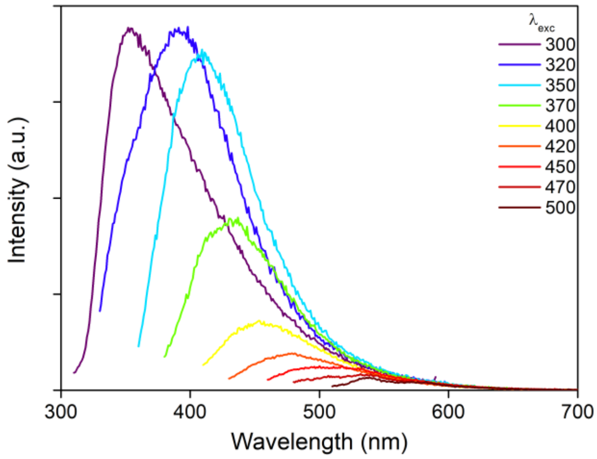

Figure S5. FL emission spectra (at different excitation wavelengths) of *a*-N-CDs with carboxylic acid groups on the surface in DMF at 298 K.

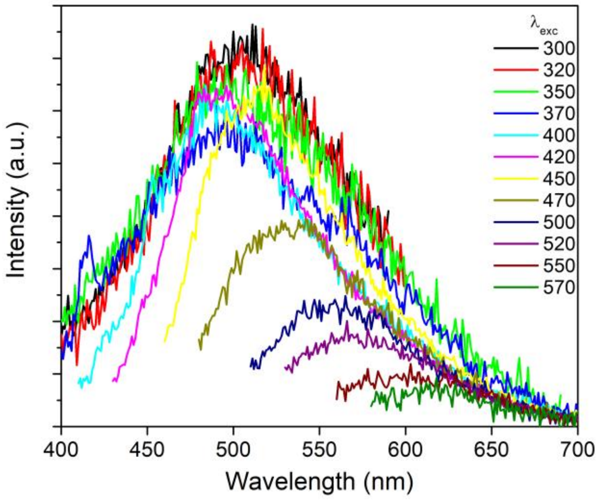

Figure S6. FL emission spectra (at different excitation wavelengths) of *g*-N-CDs with carboxylic acid groups on the surface in DMF at 298 K.

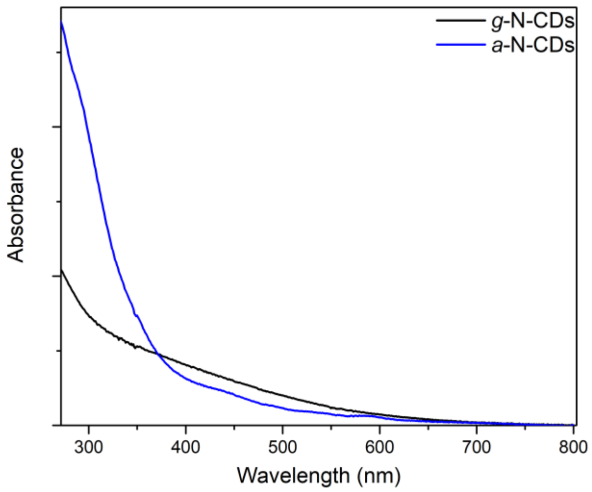

Figure S7. UV-Vis spectra of *g*-N-CDs with carboxylic acid groups on the surface (black line) and *a*-N-CDs with carboxylic acid groups on the surface (blue line) in DMF at 298 K.

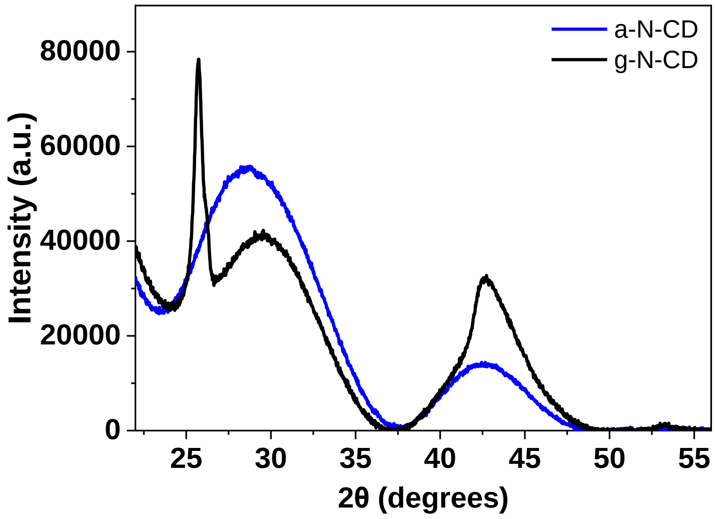

Figure S8. XRD patterns of a-N-CD and g-N-CD

Device Characterization

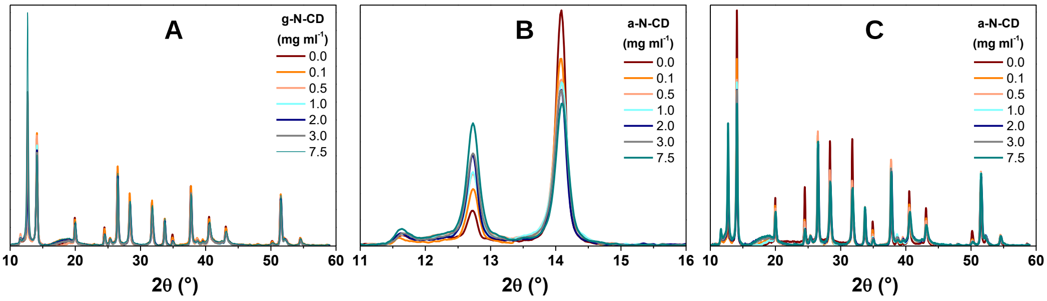

~~Figure S8~~ **Figure S9:** A) XRD analysis of the PVSK layers containing the examined concentrations of *g*-N-CDs. B) XRD analysis of the PVSK layers containing the examined concentrations of *a*-N-CDs. (Zoom) C) XRD analysis of the PVSK layers containing the examined concentrations of *a*-N-CDs.

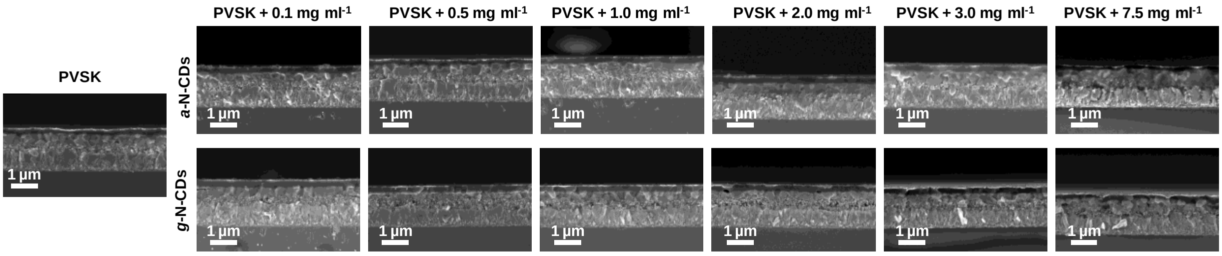

Figure S10. Cross Section FE-SEM images of PVSK containing different *a*- and *g*-N-CD concentrations.

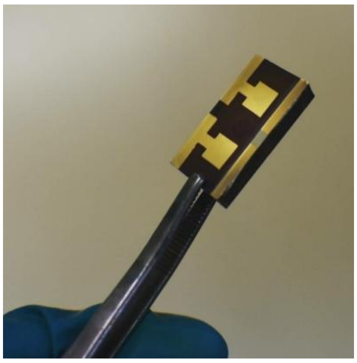

Figure S11: Picture of one of the studied devices.

Table S1: Photovoltaic parameters corresponding to the PSCs whose curves are reported in Figure 7. Approximately 10 devices were measured for each condition.

Devices	J_{SC} (mA cm^{-2})	V_{OC} (mV)	FF (%)	PCE (%)
**PVSK**	19.8	1130	76.6	16.9
**PVSK+0.1 mg ml^{-1} *g*-N-CDs**	20.3	1135	78.1	17.2
**PVSK+0.5 mg ml^{-1} *g*-N-CDs**	20.0	1136	77.5	16.8
**PVSK+1.0 mg ml^{-1} *g*-N-CDs**	19.2	1118	77.4	16.5
**PVSK+2.0 mg ml^{-1} *g*-N-CDs**	18.7	1097	75.5	15.2
**PVSK+3.0 mg ml^{-1} *g*-N-CDs**	17.6	1006	64.0	11.0
**PVSK+7.5 mg ml^{-1} *g*-N-CDs**	12.3	995	60.7	7.3

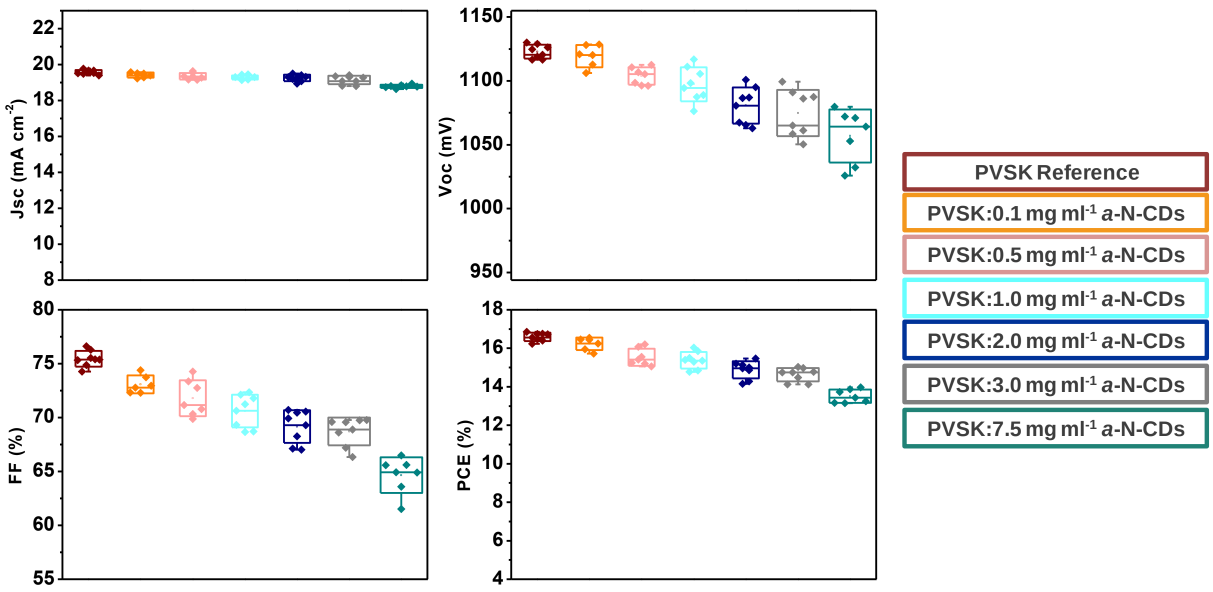

Figure S12. PV parameters for *a*-N-CD at the concentrations of 0.1, 0.5, 1.0, 2.0, 3.0, 7.5 mg ml^{-1}.

Devices	J_{SC} (mA cm^{-2})	V_{OC} (mV)	FF (%)	PCE (%)	R_s (Ω)	R_{sh} (x10^3 Ω)
**PVSK**	19.78<br>19.58 ± 0.04	1130<br>1123 ± 2	76.6<br>75.5 ± 0.3	16.9<br>16.6 ± 0.1	9.4 ± 0.6	6.4 ± 0.2
**PVSK+0.1 mg ml^{-1} *a*-N-CDs**	19.69<br>19.55 ± 0.05	1129<br>1119 ± 4	76.3<br>74.1 ± 0.7	16.6<br>16.2 ± 0.1	10.3 ± 0.6	6.0 ± 0.2
**PVSK+0.5 mg ml^{-1} *a*-N-CDs**	19.6<br>19.4 ± 0.1	1113<br>1104 ± 3	74.3<br>71.8 ± 0.6	16.2<br>15.5 ± 0.2	14.9 ± 0.9	5.2 ± 0.2
**PVSK+1.0 mg ml^{-1} *a*-N-CDs**	19.45<br>19.28 ± 0.04	1111<br>1097 ± 5	72.3<br>70.6 ± 0.5	16.0<br>15.4 ± 0.2	16.4 ± 0.8	3.8 ± 0.2
**PVSK+2.0 mg ml^{-1} *a*-N-CDs**	19.4<br>19.3 ± 0.1	1101<br>1081 ± 5	70.7<br>69.2 ± 0.5	15.2<br>14.9 ± 0.2	18.9 ± 0.7	3.0 ± 0.2
**PVSK+3.0 mg ml^{-1} *a*-N-CDs**	19.4<br>19.1 ± 0.1	1099<br>1075 ± 6	69.8<br>69.7 ± 0.5	15.0<br>14.6 ± 0.1	19 ± 1	2.7 ± 0.2
**PVSK+7.5 mg ml^{-1} *a*-N-CDs**	18.94<br>18.79 ± 0.03	1080<br>1057 ± 7	66.5<br>64.7 ± 0.6	14.0<br>13.5 ± 0.1	24.4 ± 0.7	1.7 ± 0.1

Table S2. Best and average cell parameters of the *a*-N-CD containing devices.

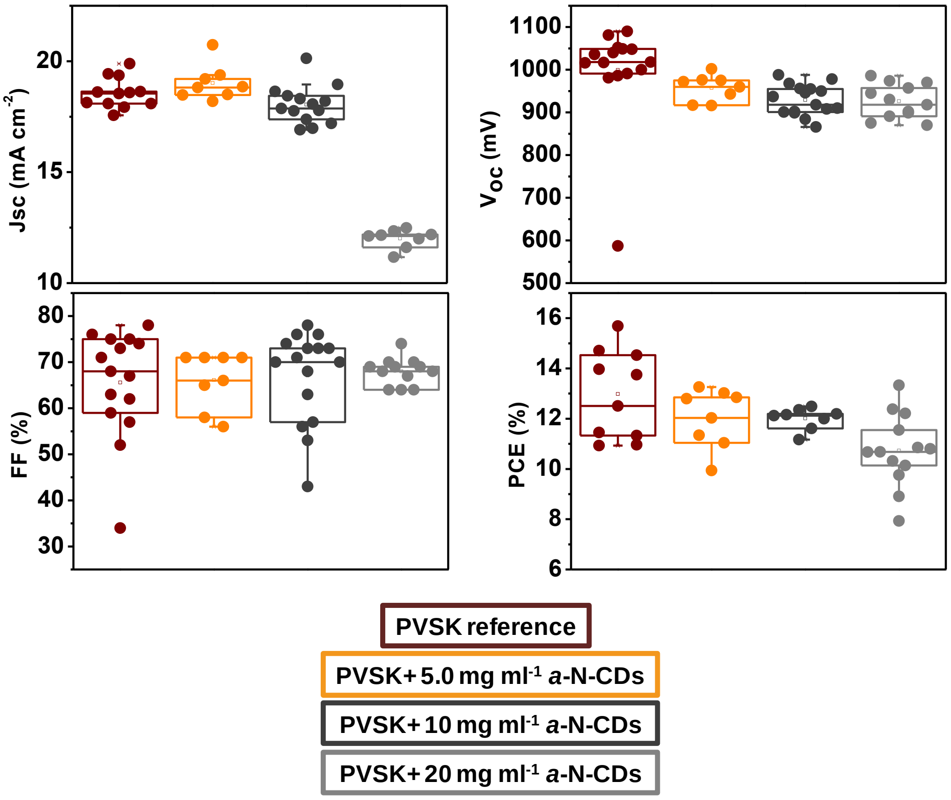

Figure S13. PV parameters for *a*-N-CD at the concentrations of 5, 10, 20 mg ml^{-1}.

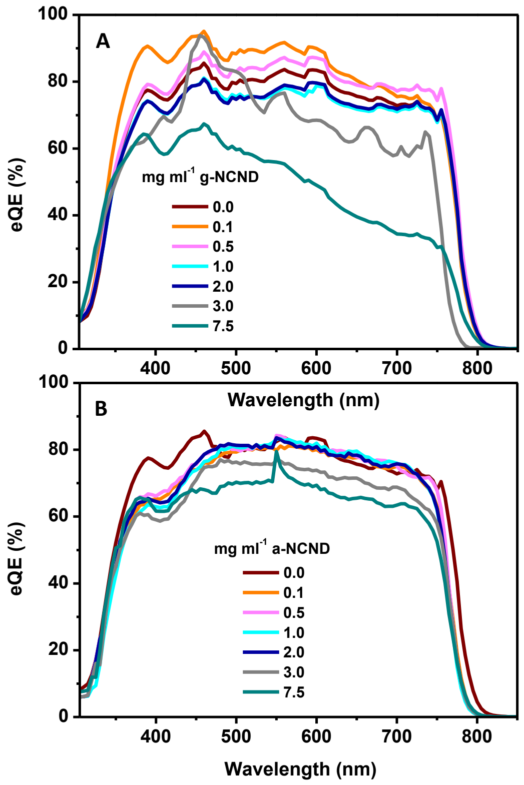

Cell (mg/ml)	*Jsc* JV (mA/cm²)	*Jsc* eQE (mA/cm²)
0.0	19.75	19.83
0.1	20.35	20.79
0.5	19.15	20.64
1.0	18.02	18.88
2.0	18.39	18.88
3.0	17.99	18.65
7.5	16.45	16.66

Cell (mg/ml)	*Jsc* JV (mA/cm²)	*Jsc* eQE (mA/cm²)
0.0	19.83	19.75
0.1	18.59	18.65
0.5	18.74	18.93
1.0	18.82	19.04
2.0	18.72	18.78
3.0	17.89	17.34
7.5	16.59	16.36

Figure S14. External Quantum Efficiency measurements (eQE) of the *g*-N-CD devices (A) and of the *a*-N-CD devices (B), together with the comparison with the J_{SC} values registered through JV curves for the same cells.

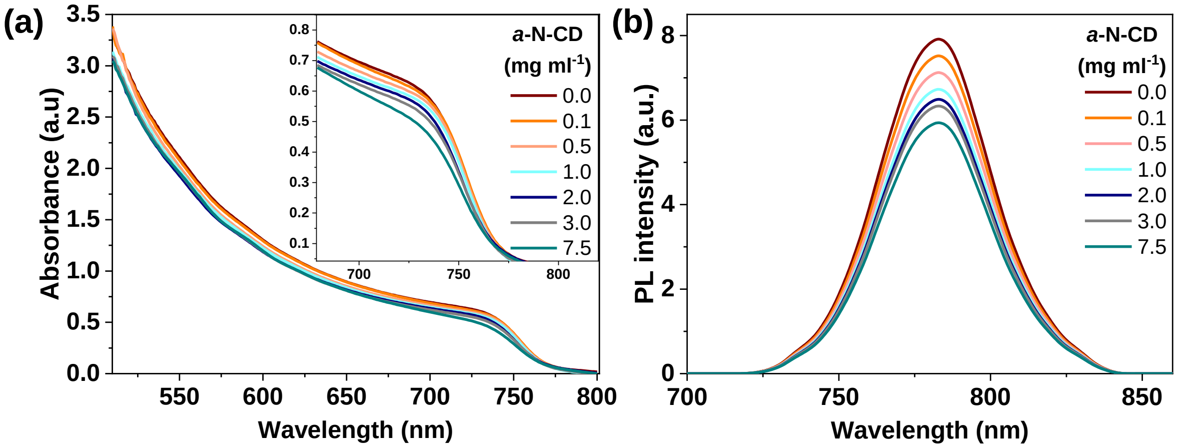

Figure S15. (a) UV–vis absorption spectra of perovskite films with different concentrations of a-N-CD additive. (b) Steady-state photoluminescence (PL) spectra with different concentrations of b-N-CD additive

Stability tests

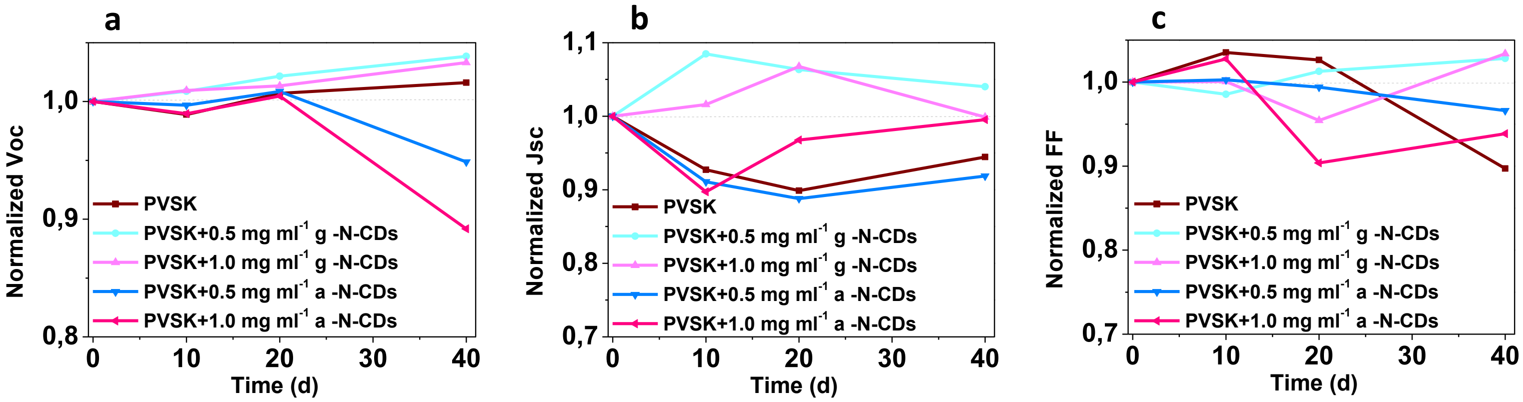

Figure S16. A) Voc, B) Jsc, C) FF obtained from long-term stability test of 0.5 and 1.0 mg mL−1 a- and g-N-CD-containing devices with respect to their PVSK reference. Three devices were tested for each condition.

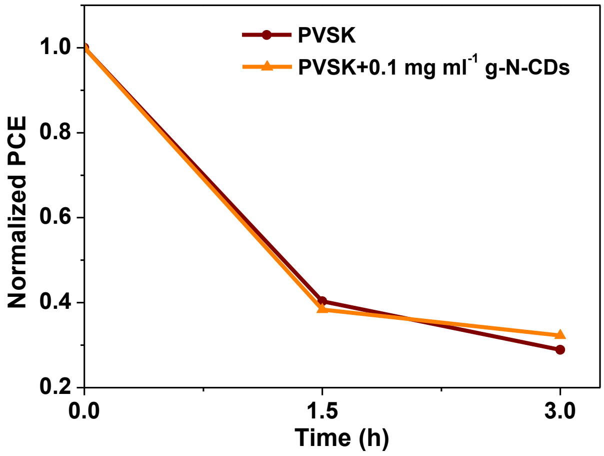

Figure S17. Thermal stress test (80 °C) under 1 sun illumination of reference device and 0.1 mg ml^{-1} *g*-N-CD containing device. 3 devices were tested for each condition.

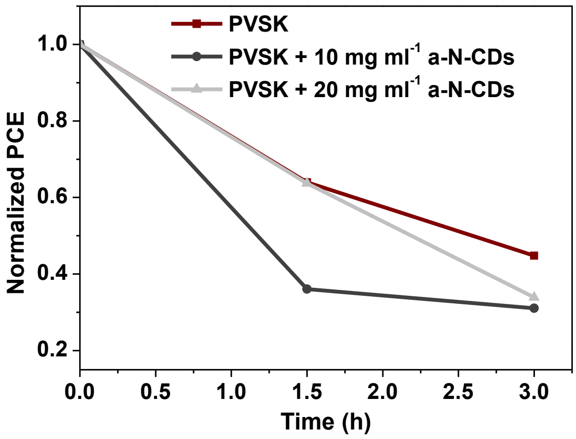

Figure S18. Thermal stress test (80 °C) under 1 sun illumination of reference device, 10 and 20 mg ml^{-1} *a*-N-CDs containing devices. 3 devices were tested for each condition.

References

[1] F. Arcudi, L. Đorđević, M. Prato, *Angew. Chem., Int. Ed.* **2016**, *55*, 2107.

[2] B. C. M. Martindale, G. A. M. Hutton, C. A. Caputo, S. Prantl, R. Godin, J. R. Durrant, E. Reisner, *Angew. Chem., Int. Ed.* **2017**, *56*, 6459.

[3] a) L. Đorđević, F. Arcudi, M. Prato, *Nat. Protoc.* **2019**, *14*, 2931;

[4] M. Saliba, J.-P. Correa-Baena, C. M. Wolff, M. Stolterfoht, N. Phung, S. Albrecht, D. Neher, A. Abate, *Chem. Mater.* **2018**, *30*, 4193.
